# Autonomous ion-highways quasi-solid electrolytes toward high-voltage lithium metal batteries

**DOI:** 10.1093/nsr/nwaf363

**Published:** 2025-08-30

**Authors:** Huajun Li, Meiying Li, Suting Weng, Jingnan Feng, Jinming Yue, Jiacheng Zhu, Kaihui Nie, Xiangzhen Zhu, Liangdong Lin, Xuefeng Wang, Huican Mao, Hong Li, Xuejie Huang, Liquan Chen, Liumin Suo

**Affiliations:** Beijing National Laboratory for Condensed Matter Physics, Institute of Physics, Chinese Academy of Sciences, Beijing 100190, China; Center of Materials Science and Optoelectronics Engineering, University of Chinese Academy of Sciences, Beijing 100049, China; Beijing National Laboratory for Condensed Matter Physics, Institute of Physics, Chinese Academy of Sciences, Beijing 100190, China; Center of Materials Science and Optoelectronics Engineering, University of Chinese Academy of Sciences, Beijing 100049, China; Beijing National Laboratory for Condensed Matter Physics, Institute of Physics, Chinese Academy of Sciences, Beijing 100190, China; Center of Materials Science and Optoelectronics Engineering, University of Chinese Academy of Sciences, Beijing 100049, China; Beijing National Laboratory for Condensed Matter Physics, Institute of Physics, Chinese Academy of Sciences, Beijing 100190, China; Center of Materials Science and Optoelectronics Engineering, University of Chinese Academy of Sciences, Beijing 100049, China; Beijing National Laboratory for Condensed Matter Physics, Institute of Physics, Chinese Academy of Sciences, Beijing 100190, China; Center of Materials Science and Optoelectronics Engineering, University of Chinese Academy of Sciences, Beijing 100049, China; Beijing National Laboratory for Condensed Matter Physics, Institute of Physics, Chinese Academy of Sciences, Beijing 100190, China; Center of Materials Science and Optoelectronics Engineering, University of Chinese Academy of Sciences, Beijing 100049, China; Beijing National Laboratory for Condensed Matter Physics, Institute of Physics, Chinese Academy of Sciences, Beijing 100190, China; Center of Materials Science and Optoelectronics Engineering, University of Chinese Academy of Sciences, Beijing 100049, China; Beijing National Laboratory for Condensed Matter Physics, Institute of Physics, Chinese Academy of Sciences, Beijing 100190, China; Center of Materials Science and Optoelectronics Engineering, University of Chinese Academy of Sciences, Beijing 100049, China; Beijing National Laboratory for Condensed Matter Physics, Institute of Physics, Chinese Academy of Sciences, Beijing 100190, China; Beijing National Laboratory for Condensed Matter Physics, Institute of Physics, Chinese Academy of Sciences, Beijing 100190, China; Center of Materials Science and Optoelectronics Engineering, University of Chinese Academy of Sciences, Beijing 100049, China; Department of Materials Science and Key Laboratory of Automobile Materials, Ministry of Education, Jilin University, Changchun 130012, China; Beijing National Laboratory for Condensed Matter Physics, Institute of Physics, Chinese Academy of Sciences, Beijing 100190, China; Beijing National Laboratory for Condensed Matter Physics, Institute of Physics, Chinese Academy of Sciences, Beijing 100190, China; Beijing National Laboratory for Condensed Matter Physics, Institute of Physics, Chinese Academy of Sciences, Beijing 100190, China; Beijing National Laboratory for Condensed Matter Physics, Institute of Physics, Chinese Academy of Sciences, Beijing 100190, China; Center of Materials Science and Optoelectronics Engineering, University of Chinese Academy of Sciences, Beijing 100049, China

**Keywords:** solid state electrolyte, lithium metal battery, high-voltage cathode, ion-percolative electrolyte

## Abstract

Electrolyte solidification holds great promise in addressing safety concerns. Nevertheless, integrating high electrochemical stability and intrinsic interfacial compatibility remains challenging for high-voltage lithium metal batteries. Herein, we report an ion-percolative quasi-solid electrolyte via concentration-driven self-assembly. At a concentration threshold (LiFSI(FEC)_x_, x = 0.37), the system triggers spontaneous crystallization of LiFSI to form a rigid, nonflammable framework at room temperature and generates dispersed [LiFSI-FEC] ionic clusters that simultaneously percolate within grain boundaries. This unique ion-percolative architecture (nano-LiFSI skeleton + [LiFSI-FEC] cluster network) enables autonomous Li-ion highways between dynamic clusters along grain boundaries of salt. The optimized electrolyte achieves a high ionic conductivity of 2.3 × 10^–4^ S/cm and an exceptional Li⁺ transference number (0.75) at room temperature. This electrolyte provides a satisfying tradeoff between nonflammability, electrochemical windows, ionic conductivity, and mechanical properties, simultaneously achieving perfect compatibility with the lithium metal anode and 4.6 V high-voltage cathodes.

## Teaser

A novel quasi-solid electrolyte design featuring concentration-driven spontaneous formation of a crystalline framework and lithium-ion highways with a percolative architecture, ensuring safety, superior ion transport, interfacial stability, and processability.

## INTRODUCTION

Lithium metal battery (LMB) technology advancements address the escalating demands for enhanced energy and power density [1–3]. Safety concerns about the combustible nature of conventional organic liquid electrolytes is being tackled by shifting toward solid-state batteries (SSBs) [4–10].

**Figure 1.**
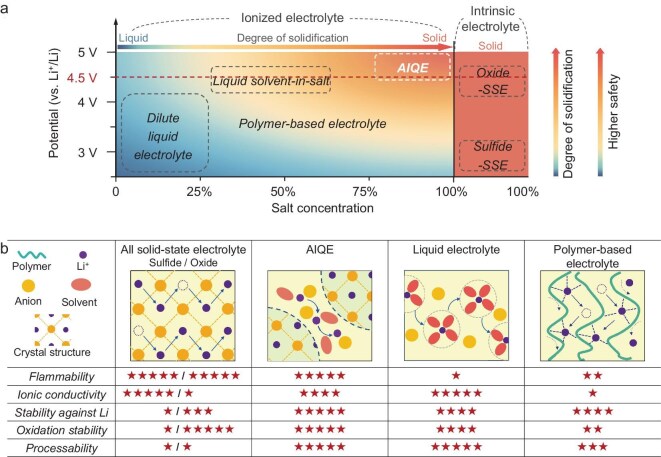
Design of the AIQE. (a) The classification of different electrolytes according to Li salt content, voltage window, and safety. (b) The physicochemical property assessment of different electrolytes.

Li-ion solid-state electrolytes (SSEs) are categorized into intrinsic and solvated Li-ion conducting electrolytes according to the Li-ion conductive mechanism, and are distinguished based on salt concentration, stable voltage range, degree of solidification, and safety, as shown in Fig. 1a. The intrinsic Li-ion conducting electrolytes (e.g. oxides and sulfides) are characterized by their homogeneous crystalline/vitreous structures where the anionic framework remains static during Li-ion migration [11,12]. They can be considered as a single-phase solute that conducts ions, with a lithium salt concentration of 100%. However, the trade-off between ionic conductivity and electrochemical stability in intrinsic SSEs stems from the inherent properties of their anionic frameworks. Generally speaking, high ionic conductivity requires weak electrostatic interactions between lithium ions and anions, whereas oxidation-reduction stability arises from the high electronegativity of anions [13–17]. For instance, the sulfide-SSEs (e.g. Li_10_GeP_2_S_12_) demonstrate high room temperature (RT) ionic conductivity comparable to liquid counterparts at 1.2 × 10^–2^ S/cm due to sulfur's high polarizability [17]. Unfortunately, their ESW is limited to the narrow range of 1.7 to 2.8 V owing to the sulfur's lower electronegativity (Fig. 1a) [18,19]. Conversely, oxide-SSEs (e.g. Li_7_La_3_Zr_2_O_12_) display a broad ESW surpassing 4 V which is due to the higher electronegativity of oxygen anions [17]. Yet their room temperature conductivities are as low as 10^–5^ S/cm ∼10^–4^ S/cm owing to the oxygen anions having lower polarizability which restricts Li-ion movement [18,20]. What's worse, these SSEs have inferior interface problems and poor processability [21–24].

Compared with the intrinsic Li-ion electrolytes, solvated Li-ion conducting solid-state electrolytes, mainly referring to polymer-based electrolytes, rely on polymer chain-mediated solvation of lithium ions [25–28]. For instance, the PEO-based electrolytes maintain flexibility to achieve good interface contact [29–32]. However, the terminal hydroxyl groups (−OH) in PEO chains are prone to oxidation above 4 V (Fig. 1a), limiting their ESW, while the high crystallinity of PEO at room temperature impedes segmental motion, thereby reducing Li⁺ mobility [33–36]. To address the interfacial challenges arising from the narrow electrochemical stability window (ESW) of polymer electrolytes, appropriate interfacial engineering strategies are desirable, such as highly efficient lithium deposition-inducing fillers, additives that enable *in-situ* formation of stable interphases, and synergistic modulation using functionalized organic frameworks [37–45].

To sum up, it is significantly challenging for the current SSE to fully meet all the requirements for commercializing LMBs, including high Li-ionic conductivity, broad ESW, manufacture-friendliness, and nonflammability. To address this, we propose a new class of ion-percolative quasi-solid electrolyte via a concentration-driven self-assembly strategy. At a critical ratio, LiFSI(FEC)_x_, x = 0.37 (>75 wt%, Fig. 1a), the liquid electrolyte transitions to a non-fluid quasi-solid state, where thermodynamically driven LiFSI crystallization forms a rigid flame-retardant framework, while percolating [LiFSI-FEC] clusters establish continuous Li⁺ transport pathways (Fig. 1b). We named this class of electrolyte as autonomous ion-highways quasi-solid electrolytes (AIQEs). This self-assembled ion-percolative architecture achieves rapid Li⁺ migration (1.73 × 10^–4^ S/cm) and a high Li⁺ transference number (0.75) via dynamic inter-cluster ion exchange. The AIQE exhibits a wide ESW (>5.5 V vs Li/Li⁺), compatible with lithium metal anodes and 4.6 V LiCoO_2_ cathodes. Pouch cells (LiCoO_2_|AIQE|Li) demonstrate superior cycling stability (60 cycles, 99.98% Coulombic efficiency) at RT. This strategy resolves the trade-off between solid-state safety and liquid-like ion transport, paving the way for commercializing high-energy-density LMBs.

## RESULTS AND DISCUSSION

### Determination of the AIQE formulation

**Figure 2.**
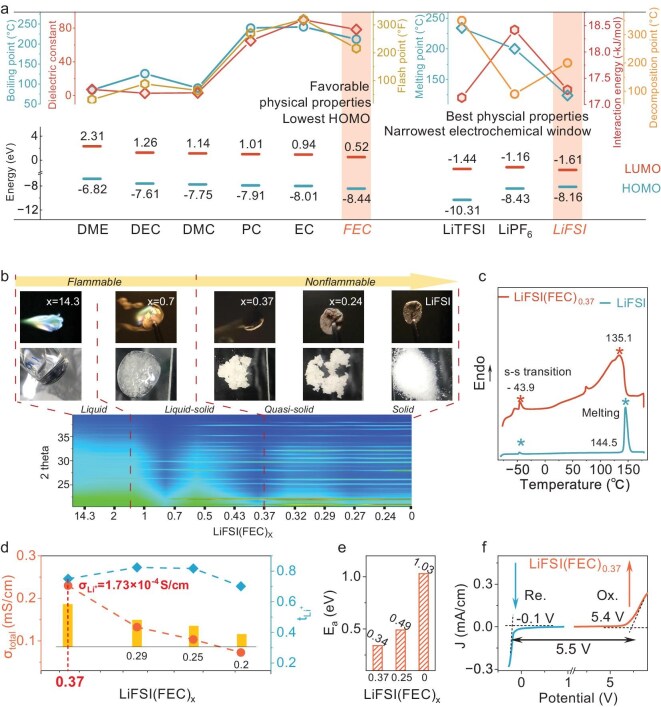
Determination of the AIQE formulation. (a) The physicochemical properties of different solvents and salts. The axes correspond to the property lines with the same colors (boiling point, dielectric constant, and flash point of solvents; melting point, interaction energy between Li-ion and anion, and decomposition point of salts). The HOMO and LUMO levels are also displayed [46,47]. (b) The XRD patterns, optical photographs, and combustion experiments of electrolytes with different LiFSI/FEC molar ratios. The whole process was carried out at RT. (c) Differential scanning calorimetry (DSC) measurement of LiFSI and LiFSI(FEC)_0.37_. (d) The ionic conductivities and Li-ion transference numbers varied with LiFSI/FEC molar ratios at RT. (e) Li-ion migration activation energy (E_a_) calculated based on the Arrhenius equation. (f) Linear sweep voltammetry (LSV) curve in a potential range of open circuit potential to 6 V and −1 V (vs Li/Li⁺) at RT, respectively.

To optimize AIQE formulation, we screened from six solvents (fluoroethylene carbonate (FEC), ethylene carbonate (EC), dimethyl carbonate (DMC), diethyl carbonate (DEC), 1,2-dimethoxyethane (DME), and propylene carbonate (PC)) and the three lithium salts (lithium hexafluorophosphate (LiPF_6_), lithiumbis(fluorosulfonyl)imide (LiFSI), and lithium bis(trifluoromethanesulphonyl)-imide (LiTFSI)). The selection followed three criteria (Fig. 2a): Criterion 1: Electrochemical Stability. The ideal solvent should exhibit the lowest HOMO level, indicating superior oxidation resistance for high-voltage cathode compatibility [46,47]. The selected salt with the narrower ESW is favorable for decomposing and forming the protective interphase [46]. Criterion 2: Dissociation Feasibility. High salt dissociation is critical to achieve AIQE. This is governed by the solvent's dielectric constant (to reduce ion pairing) and low Li⁺-anion interaction energy (to enhance dissociation kinetics). Criterion 3: Safety. The solvent should possess a high flash and boiling point to minimize flammability risks [48]. As shown in Fig. 2a, FEC and LiFSI stand out as the components of choice: FEC balances high flash point (216°C), moderate boiling point (212°C), and a large dielectric constant (78.4), while LiFSI exhibits excellent dissociation feasibility (low Li⁺-FSI^−^ interaction energy, 17.28 kJ/mol) and high thermal stability.

To determine the optimal LiFSI/FEC ratio for the AIQE, we systematically investigated concentration-dependent structural evolution and flammability through X-ray diffraction (XRD) and combustion tests (Fig. 2b, S1, and Table S1). A series of LiFSI(FEC)_x_ electrolytes were prepared via concentration-driven self-assembly: heating FEC and LiFSI to form a molten liquid followed by ambient cooling (Fig. S2), spanning from liquid to solid states. At low salt concentrations (x >1.4), LiFSI remains fully dissolved in FEC, exhibiting no LiFSI XRD peaks and high flammability for solvent-dominated composition. Increasing the salt ratio to x = 1.4, LiFSI reaches its solubility limit, initiating self-assembled crystallization of undissolved LiFSI, at which point partial XRD peaks of LiFSI appear. However, due to the low content of LiFSI crystallinity, it is still mainly in liquid form. At the critical threshold (x = 0.37), sufficient LiFSI crystallization emerges (full LiFSI XRD peaks), while eliminating flammability (Fig. S3). From this point, it comes into the AIQE electrolyte target region for nonflammability (x ≤0.37). Differential scanning calorimetry (DSC) confirmed LiFSI crystallization of the AIQE electrolyte. In the DSC curve (Fig. 2c and Fig. S4), the near-overlapping peaks of the solid–solid transition (43.9°C) and the liquidus melting transition (135.1°C) suggest the presence of crystalline LiFSI, while the broadening of the melting peak implies coexisting amorphous phases with interactions weaker than ionic bonds [49]. Subsequently, we screened the required AIQE by ion conductivity within the concentration range of x ≤0.37 (Fig. 2d, e, and Fig. S5). At the composition of LiFSI(FEC)_0.37_, the system reaches the highest Li-ion conductivity of 1.73 × 10^–4^ S/cm (σ_Li^+^_ = σ_total_ × τ_Li^+^_ = 2.7 × 10^–4^ S/cm × 0.75) together with the lowest ion transport activation energy of 0.34 eV (vs ∼1 eV of PEO-based electrolytes [50], Figs S6 and S7). Linear sweep voltammetry (LSV) analysis confirmed the electrochemical stability window of our AIQE, reaching 5.5 V (Fig. 2f)—significantly broader than that of conventional electrolytes (e.g. PEO: <4 V). In conclusion, this self-assembled AIQE (LiFSI(FEC)_0.37_, Fig. S8) reconciles nonflammability, high ion transport, and high-voltage compatibility, offering a paradigm for next-generation solid-state batteries.

### Microscopic phase structure of AIQE

**Figure 3.**
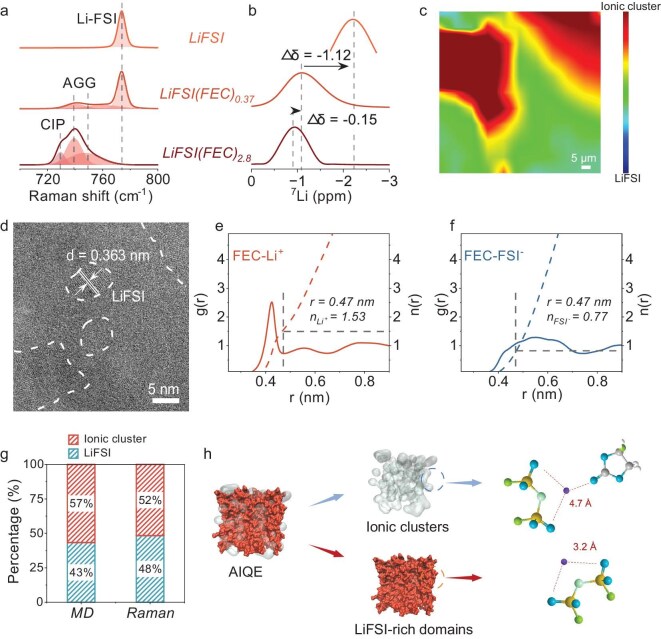
Microscopic phase structure of AIQE. (a) Raman spectra of the FSI^−^ in LiFSI(FEC)_2.8_, LiFSI(FEC)_0.37_, and LiFSI. ‘CIP’ refers to contact-ion pairs, and ‘AGG’ refers to aggregates and higher-order clusters. (b) NMR spectra of the ^7^Li chemical shift in each electrolyte. (c) Raman mapping of AIQE. The color scale represents the intensity ratio of the ionic cluster peak to the LiFSI crystal peak, with red regions indicating ionic cluster phases. (d) Cryo‐electron microscopy (Cryo-EM) image of AIQE. (e and f) Radial distribution function (RDF, left axis) and coordination number (CN, right axis) for FEC molecules to Li-ion cations and to FSI^−^ anions. (g) Phase fractions of crystalline LiFSI and ionic clusters obtained from the MD simulations and Raman mapping results. (h) Snapshot of the simulation box (10 × 10 × 10 nm^3^) of AIQE (LiFSI(FEC)_0.37_).

Building on our DSC findings that confirmed the coexistence of crystalline and amorphous phases in the AIQE, we further deciphered its dual-phase structure through spectroscopic analyses. Raman spectroscopy (Fig. 3a and Fig. S9) resolves two distinct vibrational modes: a sharp peak at 773.8 cm^−^^1^ corresponding to crystalline LiFSI and a broadened signal at 741.3 cm^−^^1^ attributed to [LiFSI-FEC] ionic clusters. The redshift of the S-N-S bond (Δ = 32.5 cm^−^^1^ vs pure LiFSI) directly evidences weakened Li⁺-FSI^−^ interactions, signifying FEC-mediated cluster formation that enhances ionic dissociation [49,51–54]. This variation in the FSI^−^ coordination environment is corroborated by Fourier transform infrared (FTIR) spectra (Fig. S10). In LiFSI(FEC)_0.37_, the S-N-S stretching vibration of pure LiFSI shifts from 760 cm^−^^1^ to 740 cm^−^^1^, and the −SO_2_ group's symmetric stretch (1200 cm^−^^1^) moves to a lower wavenumber (1179 cm^−^^1^) [55]. These results confirm that the ion dissociation phase in AIQE differs significantly from pure LiFSI. The corresponding NMR data (Fig. 3b) reveal strong upfield chemical shifts from liquid electrolyte LiFSI(FEC)_2.8_ to pure crystalline LiFSI, indicating the increased shielding of Li^+^ by FSI^−^ anions [56]. The NMR spectra of LiFSI(FEC)_0.37_ exhibits a single peak located between that of LiFSI(FEC)_2.8_ and LiFSI. This unique NMR signature confirms that Li⁺ in the AIQE resides in a hybrid coordination state: immobilized within the LiFSI lattice (crystalline phase) and dynamically moving through [LiFSI-FEC] ionic clusters (amorphous phase).

To further disclose the dual phase distribution within the AIQE, we employed multi-scale structural characterization combining Raman mapping, cryogenic electron microscopy (Cryo-EM), and molecular dynamics (MD) simulations. Raman mapping (Fig. 3c and Fig. S11) visualizes micrometer-scale phase separation, where the intensity ratio of the cluster-associated peak (741.3 cm^−^^1^) to the crystalline LiFSI peak (773.8 cm^−^^1^) transitions from blue (crystal-dominated) to brown-red (cluster-rich regions) [57]. Accordingly, the AIQE electrolyte demonstrates a heterogeneous phase distribution, and SEM imaging shows the AIQE electrolyte's clear crystalline morphology (Fig. S12). However, resolution constraints limit our observations to the micrometer scale. Cryo-EM imaging (Fig. 3d and Fig. S13) resolves anisotropic LiFSI nanocrystals (10^1^ nm grain size via Scherrer equation, Fig. S14 and Table S2) embedded in a continuous amorphous matrix—consistent with the dual-phase architecture [58]. To quantify the atomic-scale interactions, we analyzed the radial distribution functions (RDFs) of Li⁺ coordinated by FEC and FSI^−^ (Figs S15 and S16). The primary Li⁺-FEC coordination shell (0.47 nm) hosts 1.53 FEC molecules per Li⁺, while Li⁺-FSI^−^ exhibits two shells: a tight inner shell (crystalline phase) and a diffuse outer shell (cluster phase). It is confirmed that FEC preferentially solvates Li⁺, to form compact [LiFSI-FEC] clusters, while excess LiFSI nucleates into nanocrystals. To quantify the phase composition, we calculated RDFs centered on FEC molecules (Fig. 3e and f). Here we take the truncation radius (0.47 nm) of Li and FEC as the criterion, so the coordination number of Li and FSI is 1.53 and 0.77. The number of Li and FSI is 1080, and the number of FEC is 400, so the proportion of ionic clusters is 400 × 1.53/1080 = 57%, consistent with the Raman mapping results (Fig. 3g). MD snapshots (10 × 10 × 10 nm^3^, Fig. 3h) capture the phase separation dynamics in the AIQE: LiFSI preferentially coordinates with FEC to form amorphous [LiFSI-FEC] clusters, while excess LiFSI self-aggregates into energetically stable LiFSI-rich domains—a precursor to the experimentally observed crystallization. As a result, this self-assembled hierarchy evolves into a multidimensional architecture: nano grains of LiFSI connected to amorphous ionic clusters.

### Li^+^ transport mechanism of AIQE

**Figure 4.**
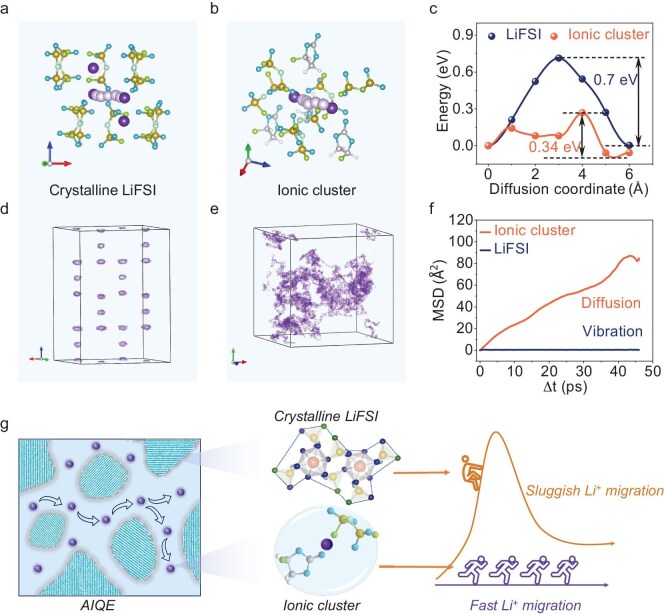
Li^+^ transport mechanism of AIQE. (a, b) Diffusion pathways of Li⁺ in crystalline LiFSI (a) and ionic clusters (b). (c) Energy barriers for Li⁺ migration in crystalline LiFSI and ionic clusters. (d, e) Trajectories of Li⁺ diffusion in crystalline LiFSI (d) and ionic clusters (e). (f) Mean squared displacement (MSD) of the two systems. (g) Schematic illustration of AIQE electrolyte structure and its ion transport mechanism.

To further understand the Li-ion transport mechanism of the AIQE, we performed nudged elastic band (NEB) and *ab-initio* molecular dynamics (AIMD) calculations. A lower energy barrier directly correlates to enhanced ionic conductivity [59]. Within the crystalline LiFSI system, robust ionic bonds constrain Li-ion migration, leading to a high energy barrier of ∼0.71 eV (Fig. 4a, c, and Fig. S17). Conversely, the LiFSI-FEC ionic cluster presents a lower activation energy of ∼0.34 eV (Fig. 4b, c, and Fig. S18), directly correlating with the experimental ionic conductivity (σ = 1.73 × 10^–4^ S/cm). To gain more insight into the Li-ion transport characteristics across AIQE systems, we conducted AIMD at RT (Fig. 4d–f). Li⁺ in crystals exhibits localized vibration (MSD <0.03 Å²/ps, Fig. 4d and f), whereas that in clusters facilitates long-range diffusion (MSD >1.5 Å^2^/ps, Fig. 4e and f) via dynamic hopping between FEC and FSI^−^. This dual phase microstructure achieves the AIQE's ion-percolation mechanism—crystalline LiFSI (43%) forms a rigid, flame-retardant framework, while amorphous clusters (57%) create interconnected pathways where Li⁺ ‘flows’ through grain boundaries via FEC-mediated hopping, akin to fluid permeation in porous media (Fig. 4g). Critically, the cluster phase's low activation energy (0.34 eV) arises from weakened Li⁺-FSI^−^ interactions, decoupling ion mobility from crystalline constraints. It is anticipated that quasi-solid electrolytes LiFSI(FEC)_x_ (x >0.37) also share similar ion transport mechanisms. However, at the critical threshold (LiFSI(FEC)_0.37_), the self-assembled biphasic architecture achieves an optimal balance: comparable crystalline (43%) and amorphous cluster (57%) proportions. Within clusters, FEC acts as dynamic solvation sites, facilitating Li⁺ hopping and enhancing diffusion [60]. Beyond this point, excessive LiFSI amplifies crystalline domains, depleting mobile Li⁺ concentration and increasing tortuosity, culminating in diminished ionic conductivity. Thus, engineering the AIQE necessitates precise control of the critical salt/solvent ratio to maximize ion-percolation pathways while suppressing crystallization-induced performance degradation.

### Electrochemical compatibility of AIQE with Li anode and LiCoO_2_ cathode

**Figure 5.**
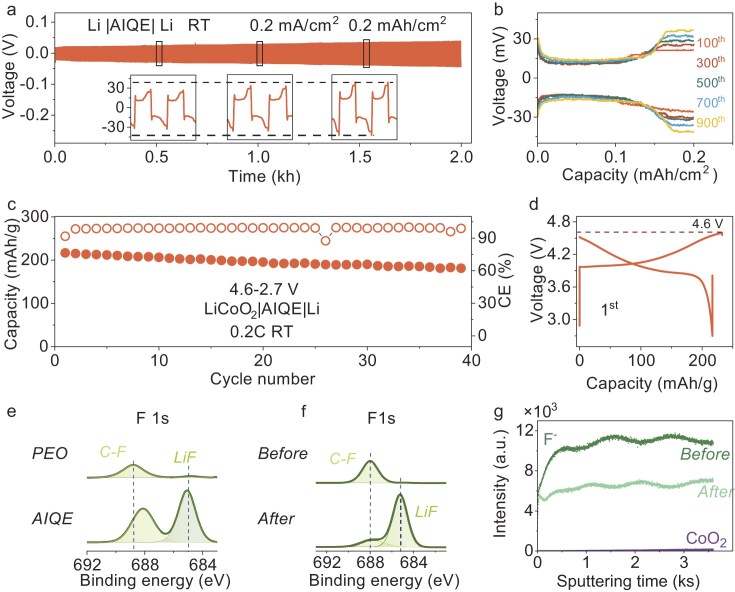
The electrochemical compatibility of AIQE with Li anode and LiCoO_2_ cathode. (a) Li plating/stripping reversibility of Li|AIQE|Li symmetric coin cell at a current density of 0.2 mA/cm^2^. (b) The overpotential of Li|AIQE|Li symmetric coin cell during cycling. (c) The cycle performance of 4.6 V LiCoO_2_|AIQE|Li coin cell. (d) The 1st charge-discharge curve of the 4.6 V LiCoO_2_|AIQE|Li coin cell. The loading of LiCoO_2_ is 2 mg/cm^2^. (e) F 1s X-ray photoelectron spectroscopy (XPS) spectra of Li anodes from PEO-based and AIQE-based LMBs, respectively. (f) F 1s XPS spectra and (g) F^−^ species depth profiles of time-of-flight secondary ion mass spectrometry (TOF-SIMS) spectra on the LiCoO_2_ cathode surface before and after cycling.

Li|AIQE|Li symmetric cells were assembled to verify the compatibility of AIQE with the Li anode. As shown in Fig. 5a, the symmetric cell reversibly deposits and strips at 0.2 mA/cm^2^ at RT, achieving excellent long-term cycling performance (2000 hours). Besides, the maximum stable cycling current can reach as high as 0.8 mA/cm^2^ (Fig. S19). Thus, AIQE demonstrates state-of-the-art performance for solid-state batteries at RT [35]. The overpotential remains below 50 mV throughout the cycling process, which is attributed to AIQE's high ionic conductivity and Li^+^ transference number (Fig. 5b) [61]. To evaluate the antioxidant performance of AIQE, 4.6 V-level LiCoO_2_ was employed. The LiCoO_2_|AIQE|Li coin cell operates in a voltage range of 2.7–4.6 V with an initial specific capacity of 210 mAh/g and delivered 40 stable cycles (Fig. 5c and d). The corresponding excellent interface stability has been confirmed by XPS analysis of the lithium surface (Fig. 5e and Fig. S20), where the SEI at the Li|AIQE interface contains higher LiF components to constrain the growth of Li dendrites [62,63]. *In-situ* EIS shows that the process of SEI gradually forms and stabilizes (Fig. S21). In addition to FEC's antioxidant properties, AIQE forms a LiF-rich cathode electrolyte interface (CEI) with exceptional stability (>6.0 V, Fig. 5f, g, Figs S22 and S23) [62,64–67]. Therefore, our AIQE electrolyte with broad ESW can be applied in high-voltage lithium metal batteries.

**Figure 6.**
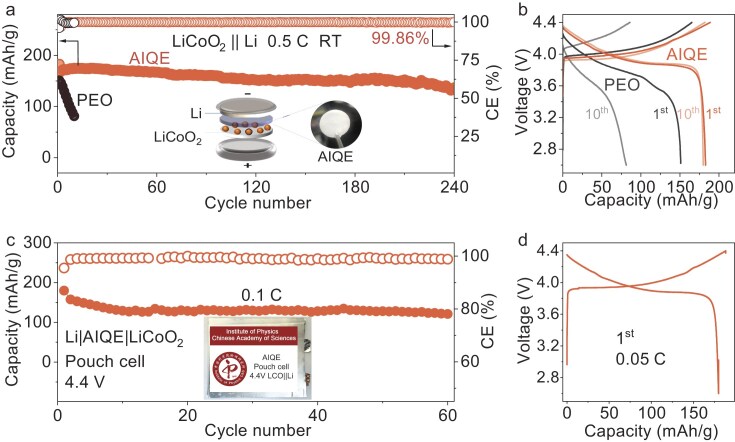
AIQE-based Li metal batteries. (a) Cycling performance of the LiCoO_2_|AIQE|Li coin cell between 2.6 and 4.4 V at RT. The LiCoO_2_ loading is 2 mg/cm^2^. (b) The 1st and 10th charge-discharge curves of the LiCoO_2_|AIQE|Li and LiCoO_2_|PEO|Li batteries, respectively. (c) Cycling performance of the LiCoO_2_|AIQE|Li pouch cell (N/P = 5.5, 20 μm Li foil, LiCoO_2_ loading is 4 mg/cm^2^) between 2.6 and 4.4 V at RT. (d) The 1st charge-discharge curves of the LiCoO_2_|AIQE|Li pouch cell.

To verify the application potential and processability of AIQE, we constructed LiCoO_2_|AIQE|Li coin cells and pouch cells, respectively. Compared to the LiCoO_2_|PEO|Li coin cell, which fails within 10 cycles due to poor high-voltage tolerance and low ionic conductivity, the LiCoO_2_|AIQE|Li coin cell delivers a high initial specific capacity (182 mAh/g) and superior cycling stability (75% capacity retention after 240 cycles, Fig. 6a and b). Moreover, the cell exhibits superior C-rate performance, with negligible capacity loss when cycled from 0.2 to 3 C and fully recovering at 0.2 C (Fig. S24). Furthermore, we assembled pouch cells that matched the AIQE with high-loading cathodes prepared by a hot-pressing method for enhancing the internal ionic conductivity of the electrode and optimizing interface contact between the electrode and electrolyte (Figs S25 and S26). Our hand-made pouch cell exhibits satisfying interfacial contact (Fig. S27) and enhanced electrode kinetics (Fig. S28). As shown in Fig. 6c and d, the LiCoO_2_|AIQE|Li pouch cell delivers 179 mAh/g capacity at the first cycle and runs for over 60 cycles with a Coulombic efficiency (CE) of up to 99.98% (Fig. S29). In this way, the outstanding processability and practicality of our AIQE have once again been confirmed (Figs S30–S32).

## CONCLUSION

In conclusion, we present a concentration-driven self-assembly strategy to engineer a high-performance quasi-solid electrolyte (AIQE). At the critical LiFSI(FEC)_x_ (x = 0.37), the AIQE self-assembles into biphasic architecture: crystalline LiFSI nano-grains (43%) providing mechanical stability and flame retardancy, and percolating [LiFSI-FEC] clusters (57%) enabling ultrafast Li⁺ migration (σ = 1.73 × 10^–4^ S/cm, τ_Li⁺_ = 0.75) via dynamic exchange. The AIQE achieves a wide electrochemical window (5.5 V), ensuring compatibility with Li metal anodes and 4.6 V LiCoO_2_ cathodes. Practical LiCoO_2_|AIQE|Li pouch cells demonstrate exceptional cycling stability (60 cycles, 99.98% CE at RT) in balancing ionic conductivity, interfacial compatibility, and processability. This work establishes ion-percolation design principles for solid-state electrolytes, where controlled phase separation circumvents the malpractice to single-phase materials. By bridging molecular-scale self-organization to macroscopic performance, the AIQE paradigm offers a scalable pathway for high-energy-density lithium metal batteries.

## Supplementary Material

nwaf363_Supplemental_File
