# Supplementary material for: Autonomous ion-highways quasi-solid electrolytes toward high-voltage lithium metal batteries
Source: Natl Sci Rev. 2025 Aug 30;12(10):nwaf363. doi: 10.1093/nsr/nwaf363 (PMC12485985; doi:10.1093/nsr/nwaf363)
Supplement: nwaf363_Supplemental_File [file nwaf363_supplemental_file.pdf]

Autonomous Ion-Highways Quasi-Solid Electrolytes Toward High-Voltage Lithium Metal Batteries

Huajun Li#[1,2], Meiying Li#[1,2], Suting Weng[1,2], Jingnan Feng[1,2], Jinming Yue[1,2], Jiacheng Zhu[1,2], Kaihui Nie[1,2], Xiangzhen Zhu[1,2], Liangdong Lin[1], Xuefeng Wang[1,2], Huican Mao[3*], Hong Li[1*], Xuejie Huang[1], Liquan Chen[1], Liumin Suo[1,2*]

1. Beijing National Laboratory for Condensed Matter Physics, Institute of Physics, Chinese Academy of Sciences, Beijing 100190, China

2. Center of Materials Science and Optoelectronics Engineering, University of Chinese Academy of Sciences, Beijing 100049, China

3. Department of Materials Science and Key Laboratory of Automobile Materials, MOE, Jilin University, Changchun 130012, China

Email: suoliumin@iphy.ac.cn, hli@iphy.ac.cn, hcmao@jlu.edu.cn

Experimental Section

Raw Materials:

Multi-walled carbon nanotubes (MWCNT) dispersed by N-Methyl-2-pyrrolidone (NMP) was purchased from Cnano. Glassfiber film was purchased from Whatman. Lithium cobalt oxide ($LiCoO_2$) material and polyvinylidene (PVDF) binder were purchased from MTI Technology Co.. Fluoroethylene carbonate (FEC), ethylene carbonate (EC), dimethyl carbonate (DMC), diethyl carbonate (DEC), 1,2-dimethoxyethane (DME) and propylene carbonate (PC) were purchased from DoDochem. Lithium hexafluorophosphate ($LiPF_6$), Lithium bis(fluorosulfonyl)imide (LiFSI) and Lithium bis(trifluoromethanesulfonyl)imide (LiTFSI) were purchased from Sdhirong. Lithium metal was purchased from Cellithium. All solvents were dried with molecular sieves before use.

Preparation of Electrolytes:

A series of LiFSI/FEC liquid electrolyte with a concentration below 15m (for example 1m, 4m, 6m, and 7m, **Table S1**) were prepared by dissolving quantitative LiFSI into FEC and stirred until completely dissolved. Solid or semi-solid electrolytes with a concentration higher than 15m (for example 15m, 20m, 30m, 40m, and 60m etc) were prepared by heating (140 ℃) and stirring a quantitative LiFSI/FEC mixture to a uniform solution and cooling at room temperature for sufficient time. For the AIQE electrolyte film, the glass fiber was cut into fixed sizes (for example Φ 16 mm and 3.5 × 3.5 cm^2) and immersed in the uniform melt formed by high-temperature stirring. After being fully immersed, it was taken out and cooled at room temperature.

Preparation of Electrodes:

The $LiCoO_2$ powder was ball milled for 40 mins at a speed of 240 r / min. The $LiCoO_2$ cathode was prepared by mixing $LiCoO_2$ powder, MWCNT (dispersed by NMP) and PVDF at a weight ratio of 93/5/2 in NMP, and then casting the slurry onto aluminum foil, which was later dried at 65 ℃ for 12 h under vacuum. The prepared $LiCoO_2$ cathode was cut into a size of Φ 12 mm or 3 × 3 cm^2, and their loading is 2~6 mg/cm^2. The lithium anode for coin cell is 800 μm thick, and that for pouch cell is 20

μm thick. which was prepared by being rolled onto copper foil. The size of that is Φ 16 mm and 3.5 ×3.5 cm^2.

Battery Assembly and Testing:

All cells were fabricated in an argon-filled glovebox and tested on battery testers (Wuhan LAND) under room temperature.

Ionic conductivity of the AIQE was measured through electrochemical impedance spectroscopy (EIS) tests by constructing an SS|AIQE|SS (SS = stainless steel) battery. The temperature and frequency were varied in the range of 0 to 70 ℃ and 10^{-2} to10^6 Hz, respectively. The Li^+ transference number ($t_{Li}{}^+$) was measured on Li|AIQE|Li symmetric cells by the chronoamperometry test. The electrochemical stability window of the AIQE was determined by a Li|AIQE|SS battery. The linear sweep voltammetry (LSV) test was conducted to evaluate the high-voltage resistance of the AIQE in a potential range of 3 to 6 V, and the low-voltage resistance of the AIQE in a potential range of 2 to -1 V at a scan rate of 0.1 mV/s. The stability of the AIQE against Li metal was estimated by symmetric Li|AIQE |Li cells at a current density of 0.2 mA/cm^2 and an areal capacity of 0.2 mAh/cm^2 at room temperature. The $LCoO_2$|AIQE |Li coin cells and pouch cells were operated between 2.6-4.4 V and 2.7-4.6 V at room temperature. The pouch cell size is 4 ×4 cm^2. The pouch cells were pretested at a rate of 0.05C for 1 cycle and then tested at a rate of 0.1C. Before assembling, the AIQE is preplaced on the cathode and subjected to a certain pressure (under the gravity of a 0.8 cm thick steel plate) at a temperature of 60 ℃ for a certain period (about 3 hours) to allow the ion transport channel to penetrate the cathode inside.

Material Characterizations:

Thermo-gravimetric analysis (TGA，STA 449 F5 Jupiter) was performed ranging from30 to 400 ℃ at a heating rate of 10 ℃/min. The differential scanning calorimetry (DSC, TA NETZSCH) was conducted ranging from -80 to 180 ℃ at a heating rate of 10 ℃/min. Solid state nuclear magnetic resonance (ss-NMR) data were collected on a Bruker Avance NEO 600 MHz at room temperature with 1 M LiCl D_2O used as the reference. Scanning electron microscope (SEM) data were collected on a Regulus

8100, operated at 10 kV. X-ray diffraction (XRD) data were collected on a Rigaku Smart-Lab 9Kw. Cyclic voltammetry (CV) and linear sweep voltammetry (LSV) data were all collected on a CHI660E and an Autolab PGSTAT302N at a scan rate of 0.1 mV/s. Electrochemical impedance spectroscopy (EIS) data were collected on the same equipment. The X-ray photoelectron spectroscopy (XPS) spectra were performed by ESCALAB 250 with monochromatic Al kα radiation. The Raman spectra of the electrolytes were measured with an NRS-5100 spectrometer (JASCO) between 200 and 2000 cm^{-1}. The nanoscale structure was collected by Cryo - electron microscopy (Cryo-EM, JEOL JEM-F200). The depth composition distribution was measured by time-of-flight secondary ion mass spectrometry (TOF-SIMS).

Molecular Dynamics Simulation:

MD package GROMACS[1] was used to carry out the molecular dynamics (MD) simulations. The model was constructed with 1080 Li, 1080 FSI ions and 400 FEC molecules. The force field parameters for Li^+ and FSI^- were obtained from Jensen et al[2]. and Gouveia et al.[3], respectively. The force field parameters of FEC molecule was generated by LigParGen web server[4], except for its restrained electrostatic potential (RESP) atomic partial charges, which were assigned to each atom based on the electrostatic potential (ESP) charges obtained from the Multiwfn program[5]. These molecules and ions were initially packed randomly in a cubic box of size 10 × 10 × 10 nm^3 using Packing Optimization for Molecular Dynamics Simulations (PACKMOL)[6] .

The steepest descent with a convergence criterion of 500 kJ mol^{-1} nm^{-1} and Berendsen barostat[7] were employed to minimize and equilibrate the initial configuration, respectively. Subsequently, the model was heated from 298 K to 700 K and maintained at 700 K for 2 ns, and subsequently annealed from 700 K to 298 K. Finally, the production runs of 500 ns in NPT ensemble under Nóse-Hoover thermostat[8, 9] and Parrinello-Rahman barostat[10] were conducted at 298 K and 1 bar. The last 10 ns simulations were used for the radial distribution function (RDF) analysis. The van-der-Waals interactions was calculated with a cutoff distance of 1.2 nm. The

particle-mesh Ewald (PME)[11] method with a 1.2 nm real space cut-off was used to calculate the electrostatic interactions.

Density Functional Theory (DFT) Simulations:

The Gaussian 16 program was used to calculate the HOMO and LUMO levels of FEC, EC, PC, DEC, DMC and DME molecules, and $LiPF_6$, LiFSI and LiTFSI salts. In the calculations, geometry optimization was conducted using Lee-Yang-Parr correlation functional (B3LYP) [12]at 6-311+G (d,p) level.

The nudged elastic band (NEB)[13] and Ab-initio molecular dynamics (AIMD) calculations were carried out employing the Vienna Ab-initio Simulation Package (VASP) code based on density functional theory[14, 15]. The exchange-correlation potential is described by the generalized gradient approximation (GGA) in the Perdew-Burke-Ernzerhof (PBE) form[16]. For these DFT calculations, energy cutoff of 500 eV, and energy and force convergence criterion of 10^{-5} eV and 0.01 eV/ Å were employed. The structure of crystalline LiFSI was built based on its crystalline structure with P63/m space group, and a = b = 8.74 Å, c = 12.97 Å. Liquid LiFSI-FEC structures were constructed by packing randomly 12 Li^+, 12 FSI^-, 8 FEC in a cubic box of size 22×22×22 $Å^3$. The AIMD simulations were carried out at 298 K, lasting for 50000 steps with a time step of 1 fs. The first 4 ps are used to equilibrate the system, and the mean-squared displacement (MSD) was calculated for the last 46 ps. For NEB calculations, the initial structure was constructed based on the structure after AIMD simulations.

Navigation

For Fig. 2: Fig. S1, S2, S3, S4, S5, S6, S7 and S8; Table S1

For Fig. 3: Fig. S9, S10, S11, S12, S13, S14, S15 and S16; Table S2

For Fig. 4: Fig. S17 and S18

For Fig. 5: Fig. S19, S20, S21, S22 and S23

For Fig. 6: Fig. S24, S25, S26, S27, S28, S29, S30, S31, and S32.

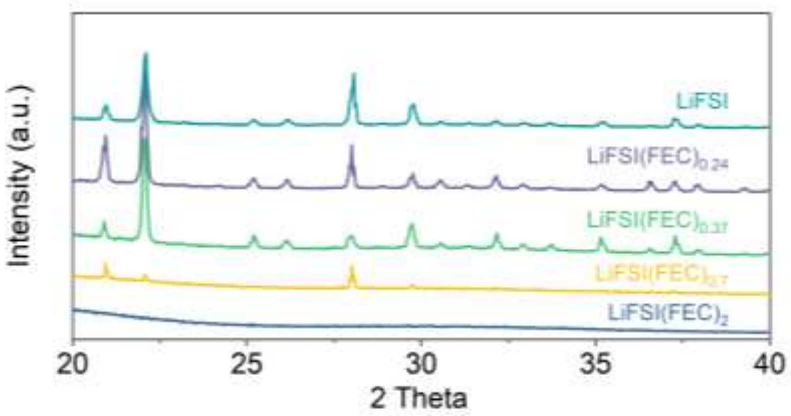

Figure S1. The XRD spectra of the electrolyte at different concentrations.

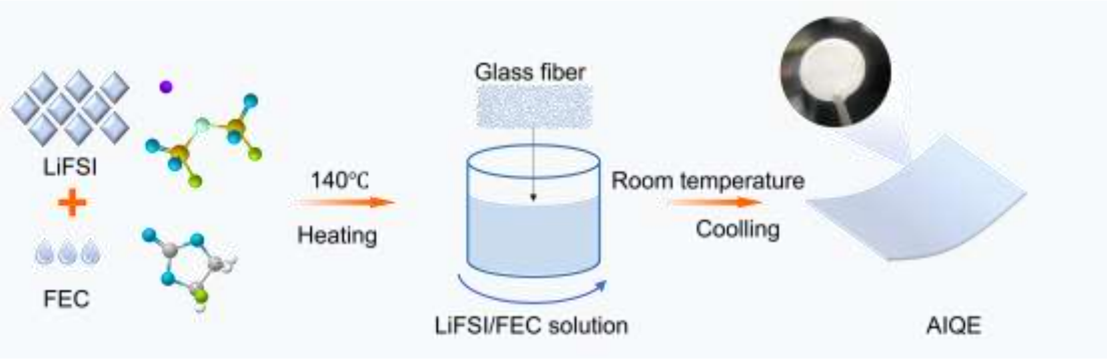

Figure S2. The preparation of AIQE.

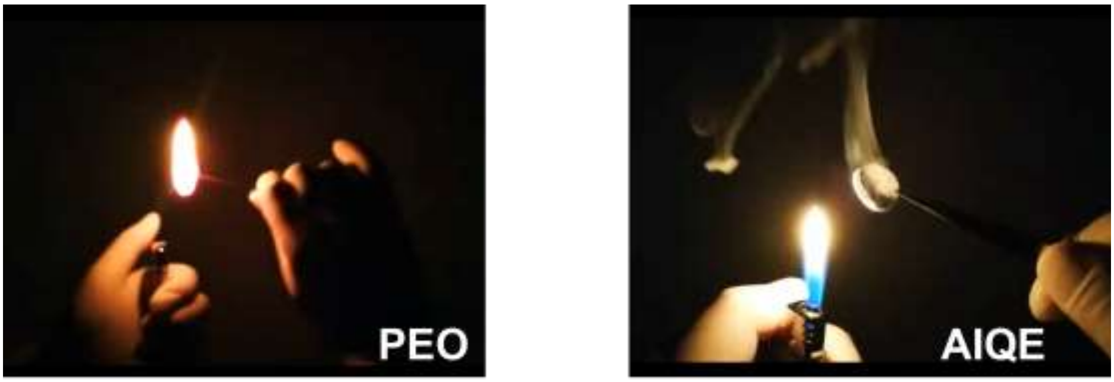

Figure S3. Comparison of combustible experiments between PEO electrolyte and AIQE electrolyte.

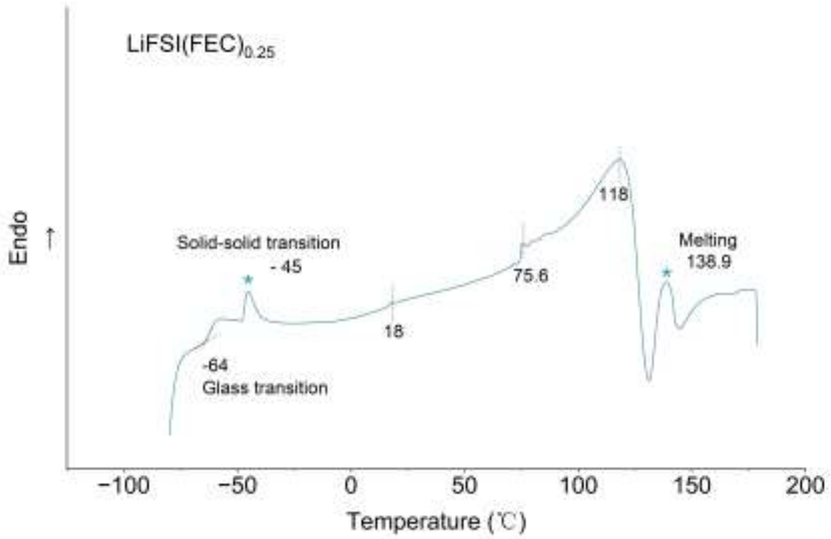

Figure S4. The differential scanning calorimetry (DSC) measurement of $LiFSI(FEC)_{0.25}$.

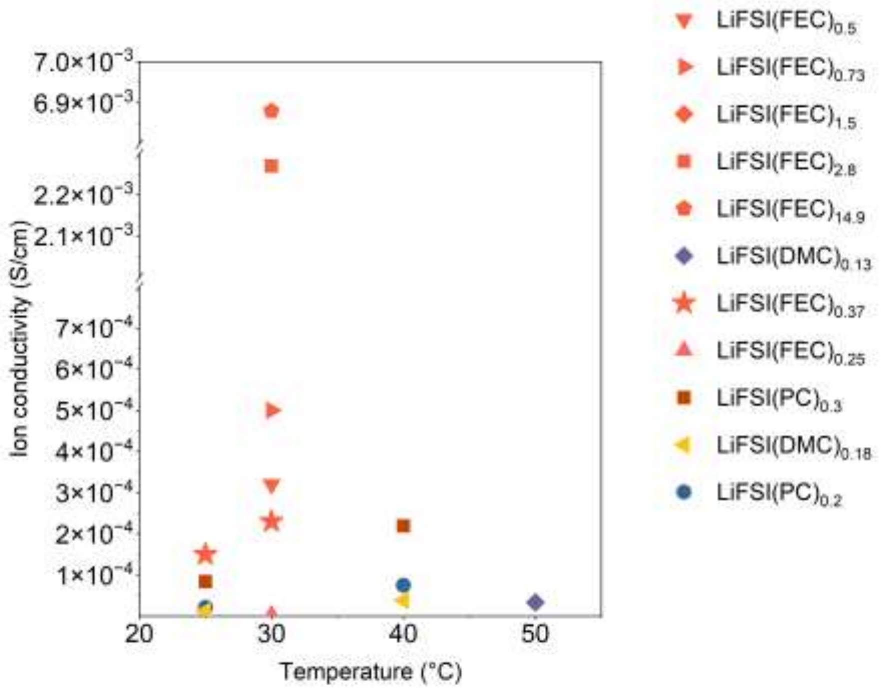

Figure S5. Comparison of AIQE-type electrolytes ion conductivity with different formulations.

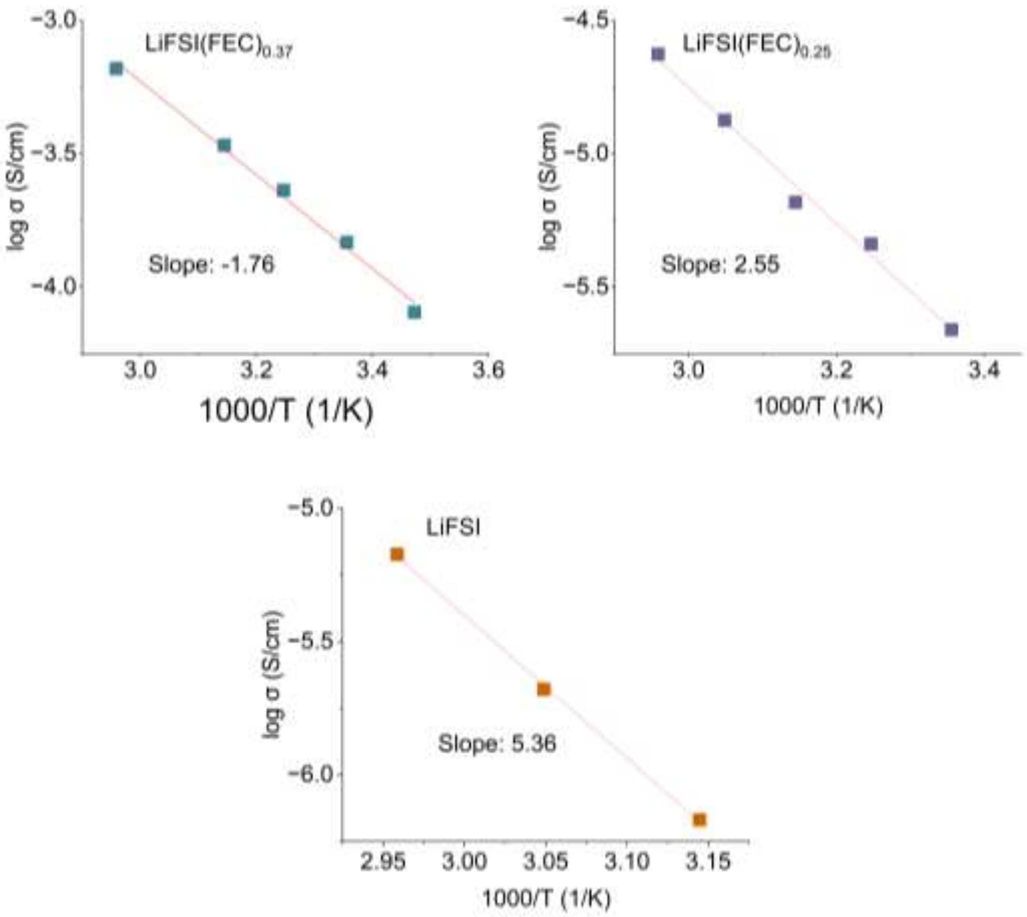

Figure S6. Arrhenius plot of the LiFSI(FEC)$_{0.37}$, LiFSI(FEC)$_{0.25}$, and LiFSI.

The activation energy for lithium-ion transport can be calculated using the following formula[17]:

$$\sigma = A\exp(-\frac{E_a}{RT}) \quad (1)$$

$$\log\sigma = \log A - \frac{E_a}{2303R}(\frac{1000}{T}) \quad (2)$$

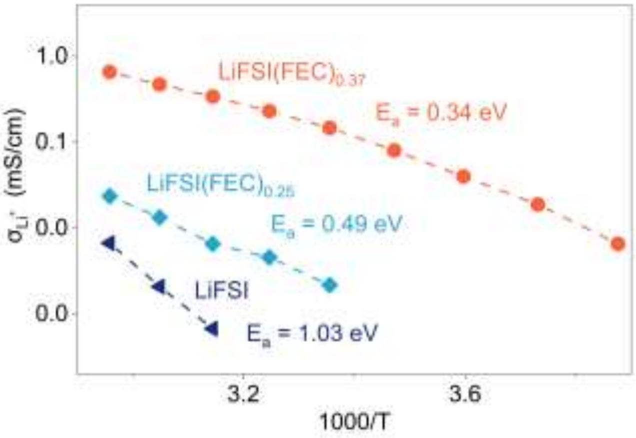

Figure S7. Temperature dependent Li-ion conductivity curves of AIQE ($LiFSI(FEC)_{0.37}$ and $LiFSI(FEC)_{0.25}$) and pure LiFSI electrolytes. Li-ion migration active energy (Ea) calculated based on the Arrhenius equation.

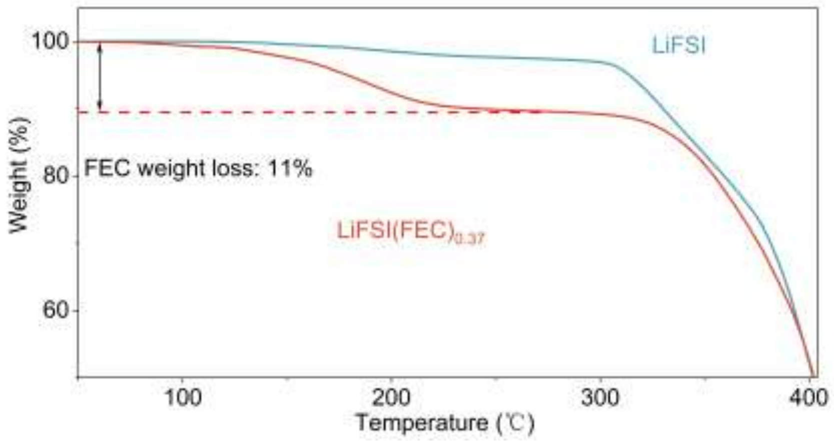

Figure S8. The thermogravimetry (TG) measurement of $LiFSI(FEC)_{0.37}$ shows the FEC content of 11 wt.%.

The TG curve indicates a sharp decline in the mass of AIQE between 100 ℃ and 200 ℃, stabilizing after that. Given that FEC has a boiling point of 210 ℃, it can be determined that the FEC content in AIQE is about 11 % by weight, aligning with our experimental design.

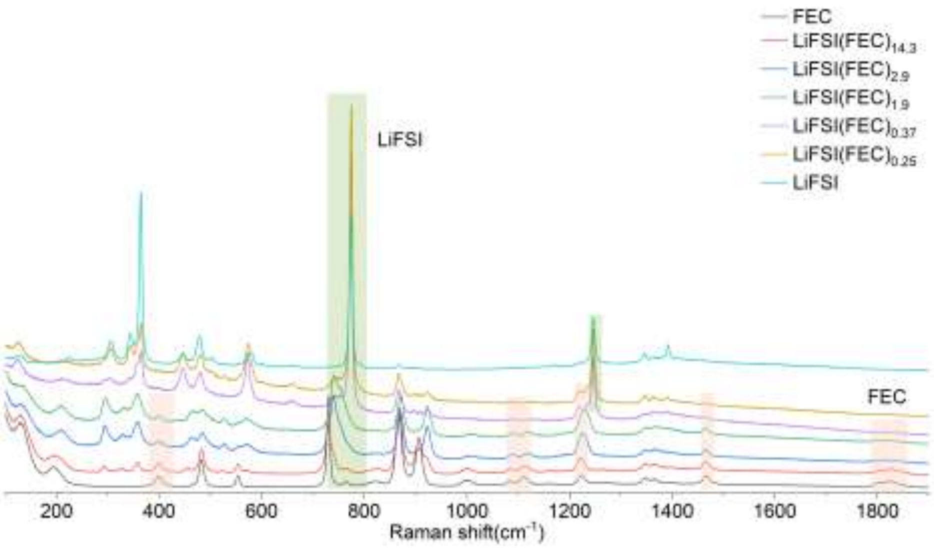

Figure S9. Full Raman spectra of electrolyte with different LiFSI/FEC mole ratio.

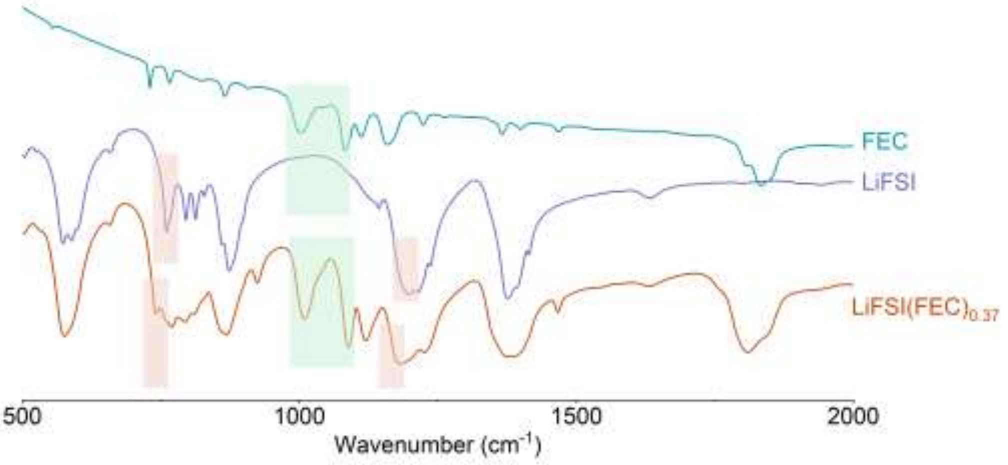

Figure S10. Fourier transform infrared (FTIR) spectra of the $LiFSI(FEC)_{0.37}$, FEC, and LiFSI.

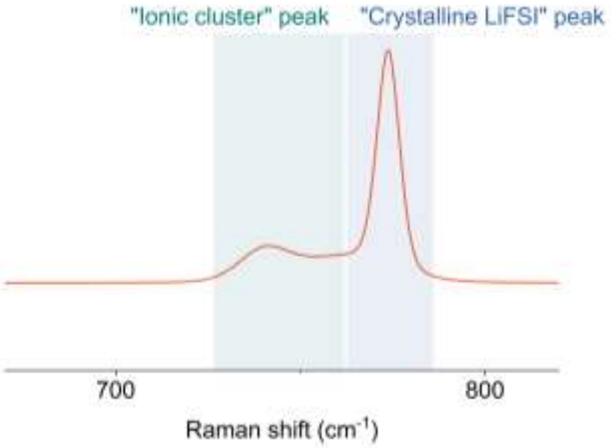

Figure S11. Two peaks corresponding to Raman mapping intensity bar.

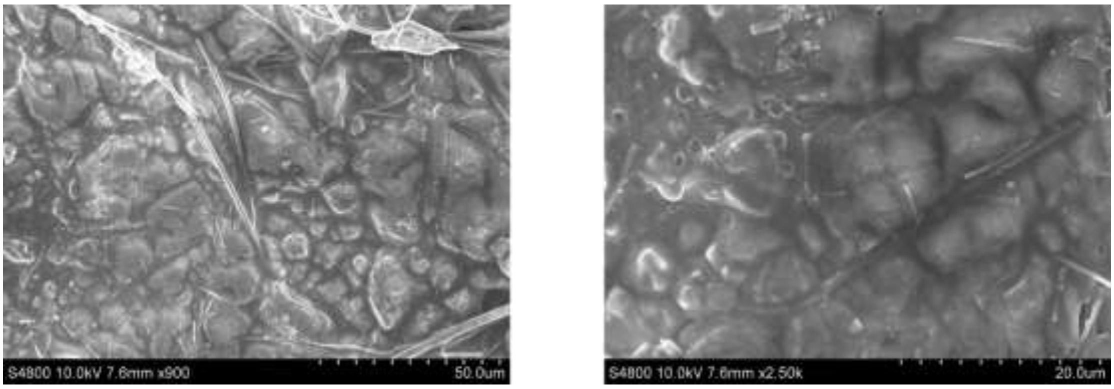

Figure S12. SEM image of AIQE.

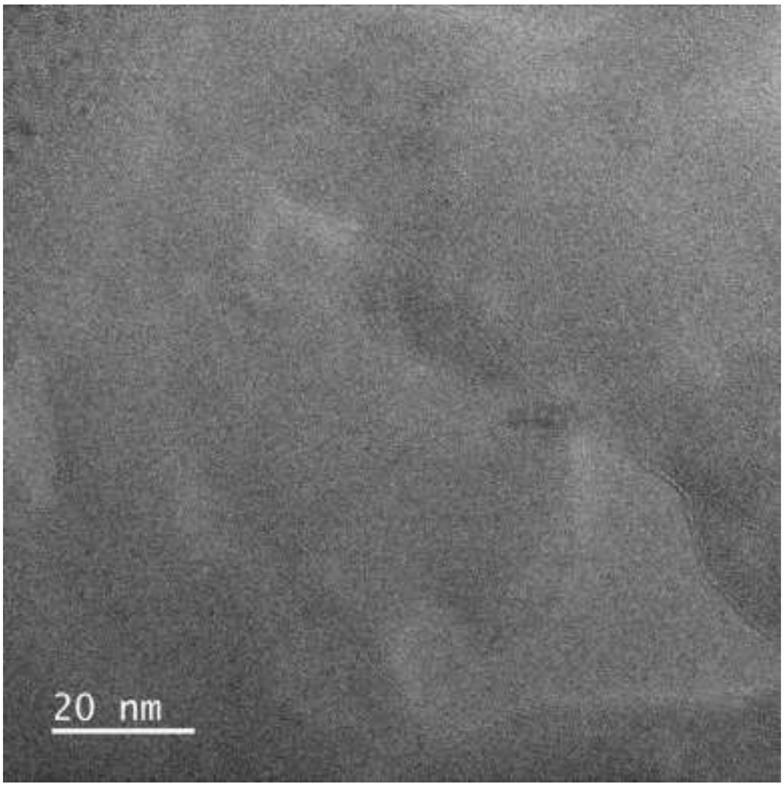

Figure S13. Cryo-EM image of AIQE.

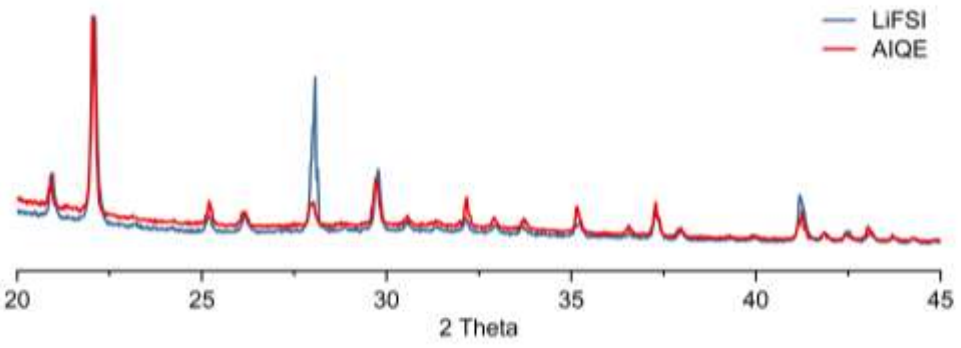

Figure S14. XRD spectra of AIQE.

Scherrer equation：

$$\tau = \frac{K\lambda}{\beta \cos \theta} \quad (3)$$

τ: Mean size of the ordered grains.

K: Dimensionless shape factor - 0.9.

λ: X-ray wavelength.

β: Full line width at half the maximum intensity (FWHM).

θ: Bragg angle.

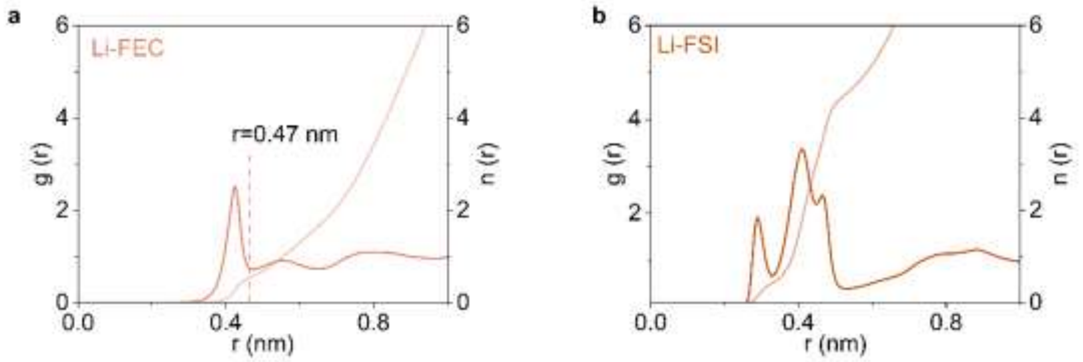

Figure S15. RDF and CN calculated between Li^+ and FEC molecules (a) and FSI^-(b).

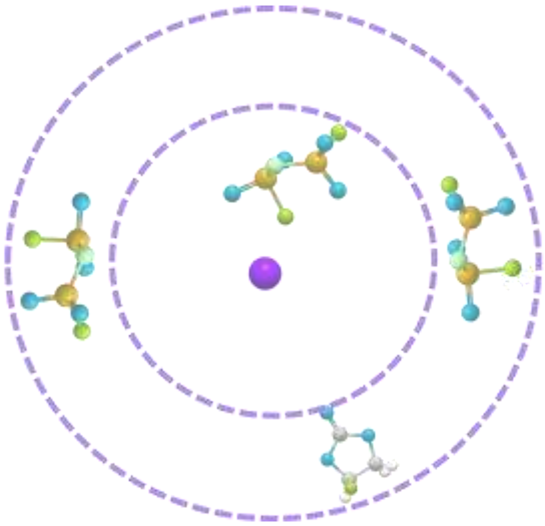

Figure S16. Scheme of ion aggregation clusters based on RDF and CN.

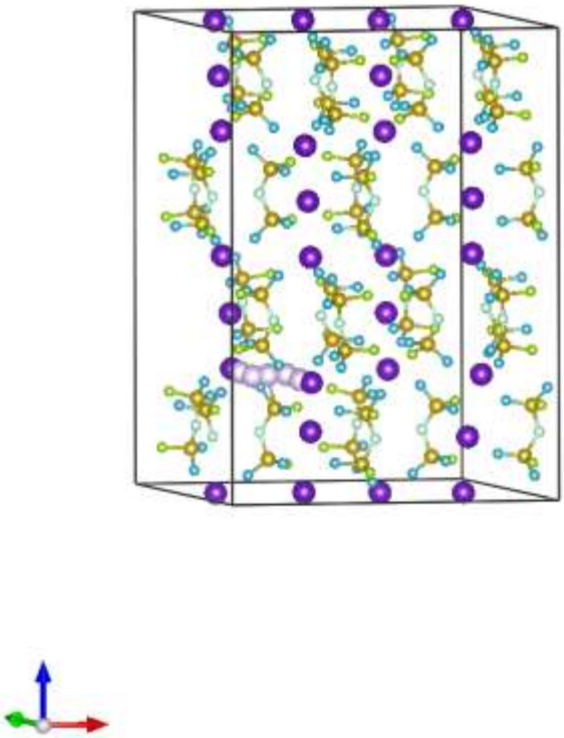

Figure S17. Diffusion paths of Li atom in crystalline LiFSI.

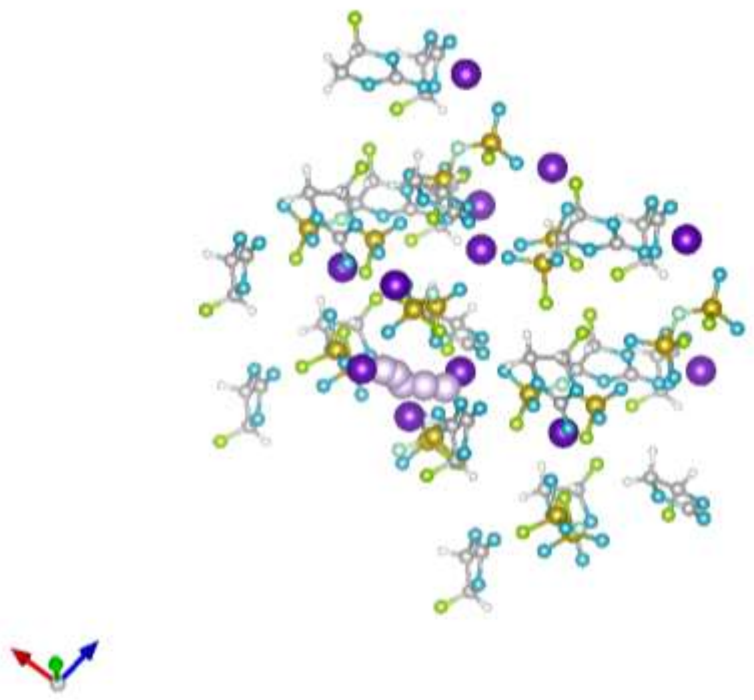

Figure S18. Diffusion paths of Li atom in [LiFSI-FEC] ionic cluster.

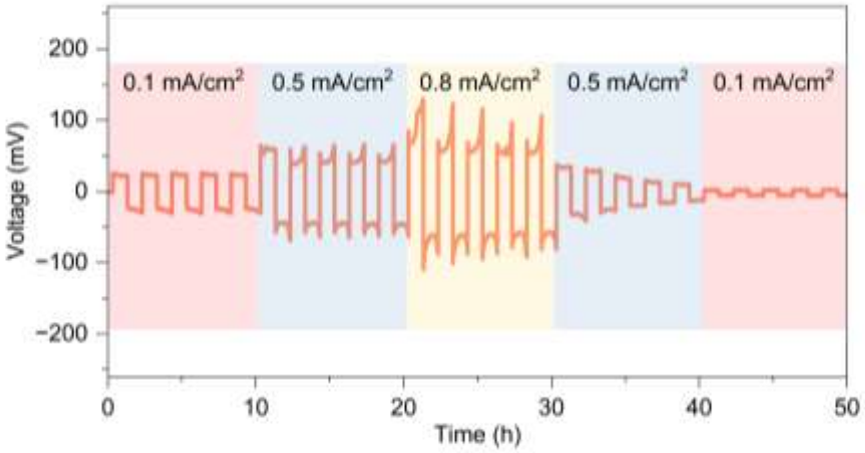

Figure S19. Li plating/stripping reversibility of Li|AIQE|Li symmetric cells at various current densities.

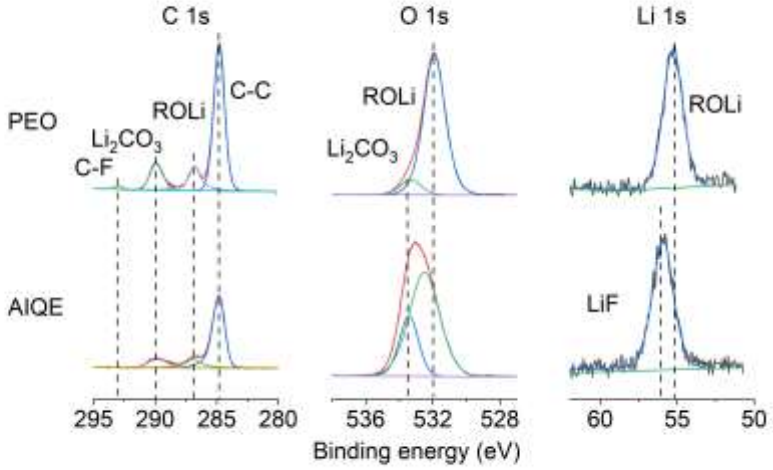

Figure S20. XPS analysis of Li anode from PEO-based and AIQE-based LMBs.

There is high interfacial energy between LiF and Li metal, which can ensure sufficient mechanical strength during the Li plating/stripping process without causing SEI fracture due to significant volume changes, promoting the planar diffusion of Li-ion, and constraining the growth of lithium dendrites.

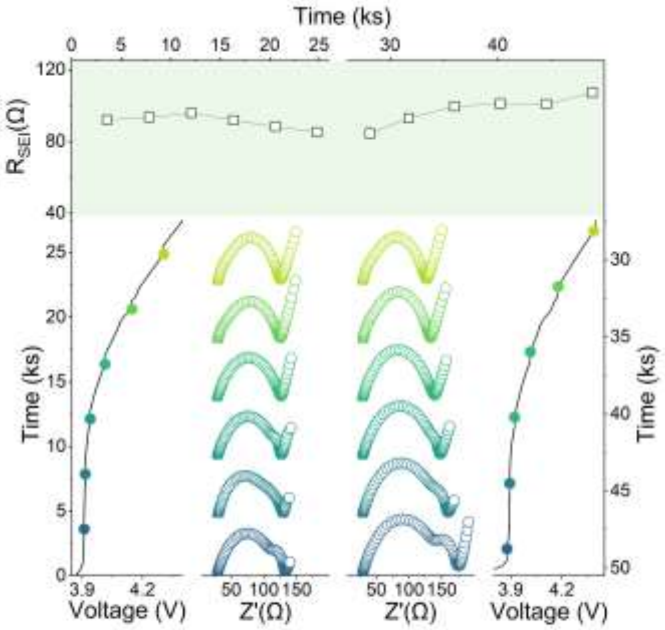

Figure S21. In situ EIS of the $LiCoO_2$|AIQE|Li coin cell.

In situ EIS shows that the impedance of SEI (R_{SEI}) increases first, then decreases, and finally stabilizes during the 1st charging and discharging process, indicating the gradual formation of stable SEI at the anode interface.

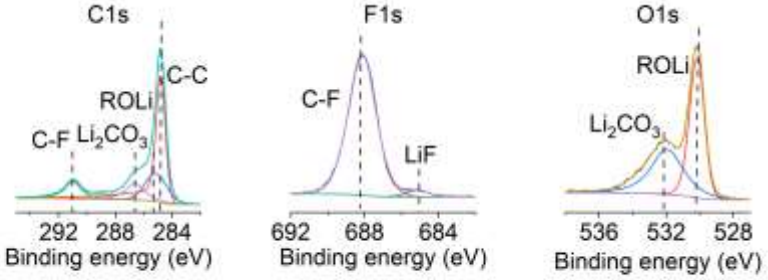

Figure S22. XPS analysis of $LiCoO_2$ cathode from $LiCoO_2$|PEO|Li coin cells.

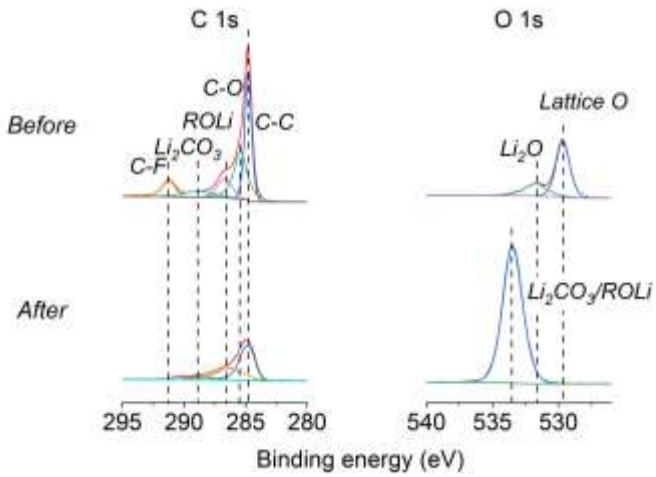

Figure S23. C1s and O1s spectra of XPS analysis of $LiCoO_2$ cathode from the $LiCoO_2$| AIQE|Li coin cell before and after cycling.

The LiF signal (684.6 eV) appears on the cycled $LiCoO_2$ cathode surface, significantly more potent than the C-F signal (688.8 eV), resulting from the decomposition of FSI^- anions. The depth distribution of TOF-SIMS indicates that the CEI of $LiCoO_2$ cathode is mainly composed of inorganic F^- fragments, consistent with XPS results. the dense and robust CEI, passivating the surface and inhibiting the further occurrence of parasitic reactions between the high-voltage $LiCoO_2$ cathode and the electrolyte.

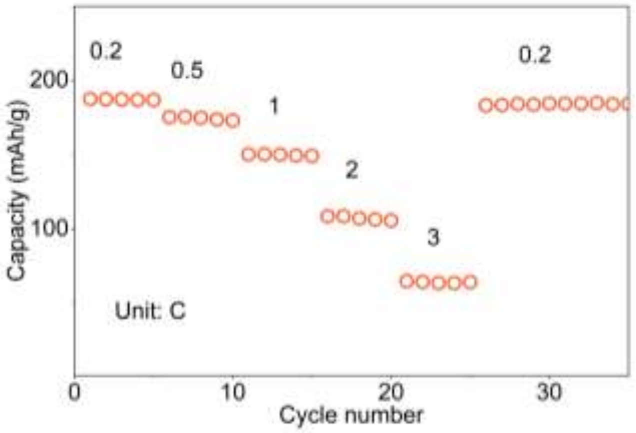

Figure S24. C-rate of $LiCoO_2$|AIQE|Li battery.

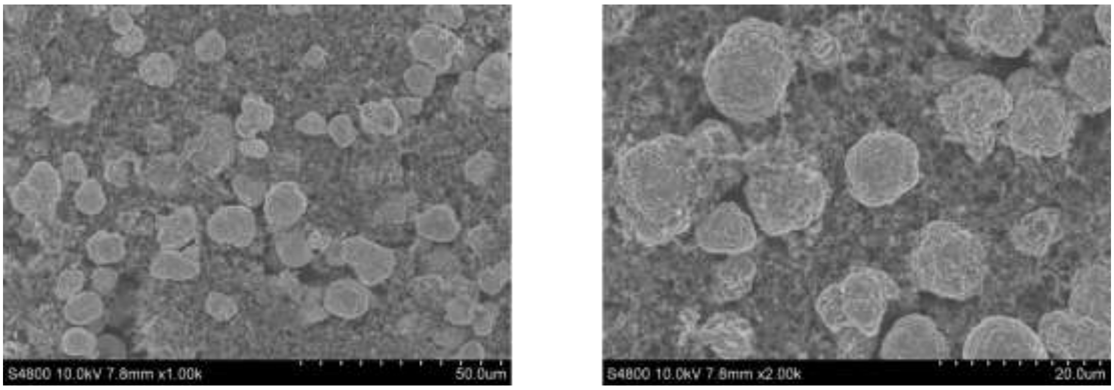

Figure S25. SEM images of the pristine $LiCoO_2$ cathode.

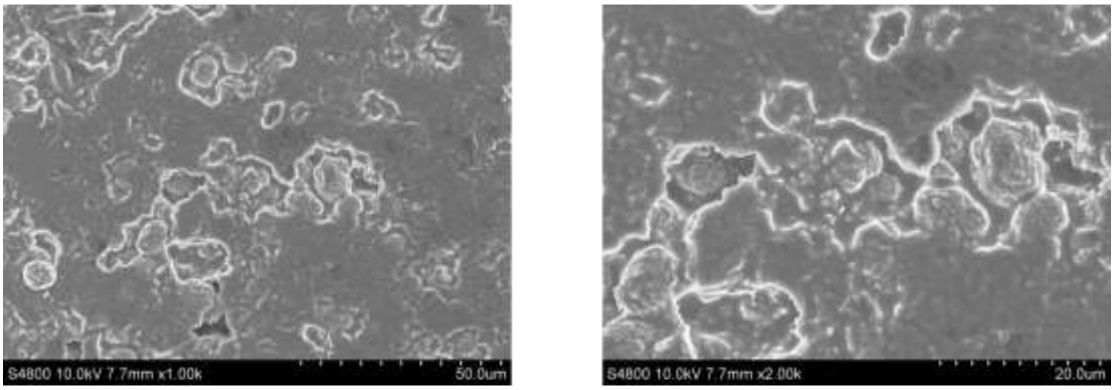

Figure S26. SEM images of the processed $LiCoO_2$ cathode by hot-pressing method.

Before cell assembly, we placed the AIQE electrolyte on the cathode at 60 ℃ under a certain pressure for a set time, resulting in a cathode with ideal ionic pathways.

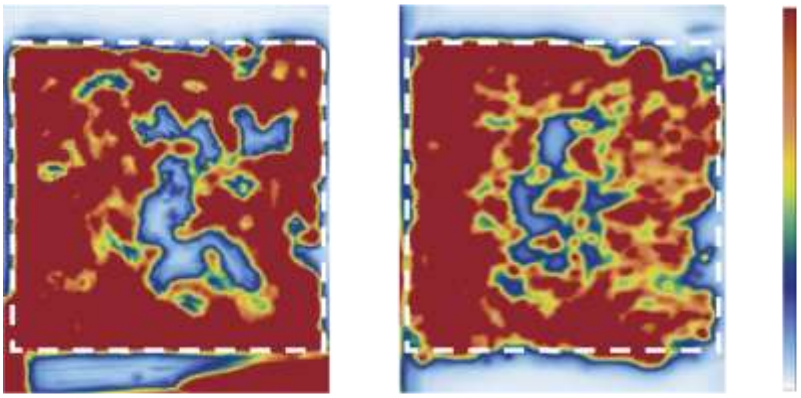

Figure S27. Ultrasonic transmission images of $LiCoO_2$|AIQE|Li pouch cell before (left) and after (right) 60 cycles.

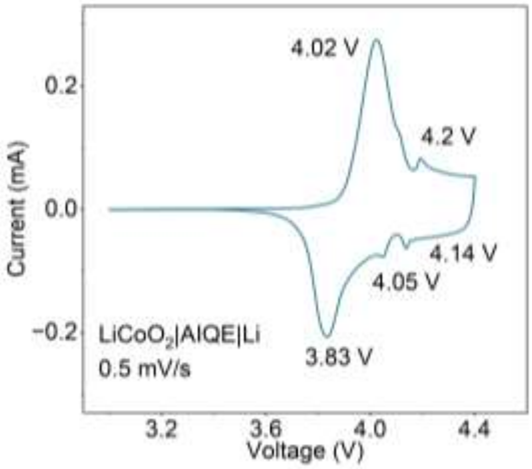

Figure S28. CV curve of $LiCoO_2$|AIQE|Li coin cell at a scan rate of 0.5 mV/s.

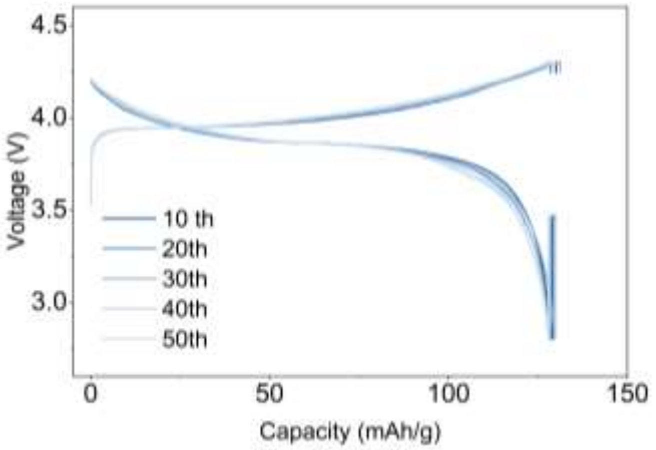

Figure S29. Charge-discharge curves of the $LiCoO_2$|AIQE|Li pouch cell.

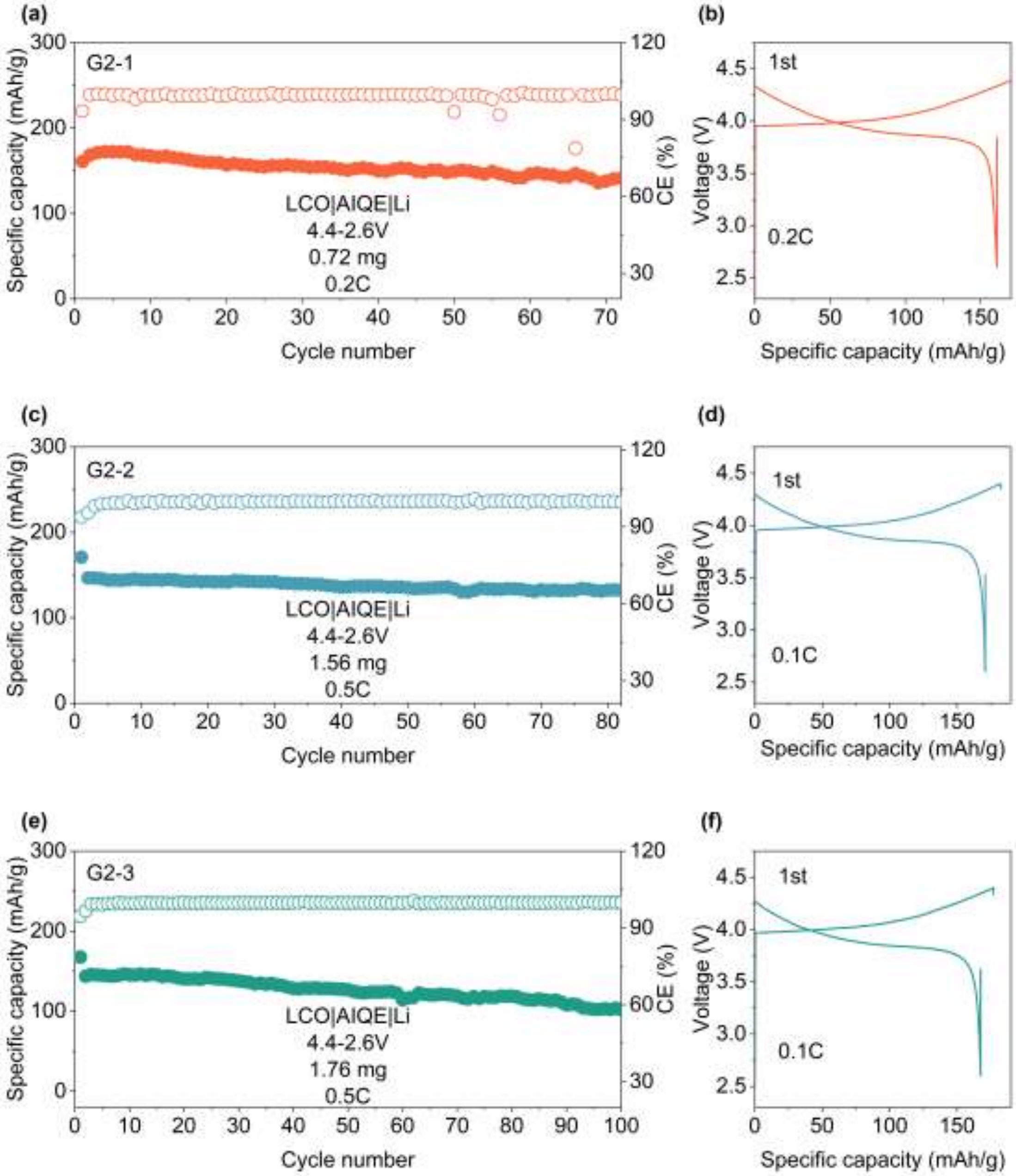

Figure S30. The cycling performance of LCO|LiFSI(G2)$_{0.185}$|Li batteries under various cathode mass loadings and C rates.

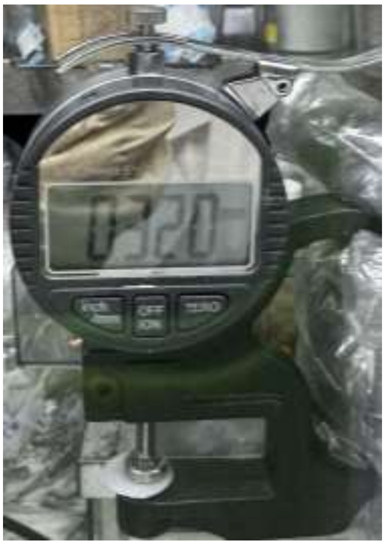

Figure S31. The typical thickness of AIQE measured by a thickness gauge.

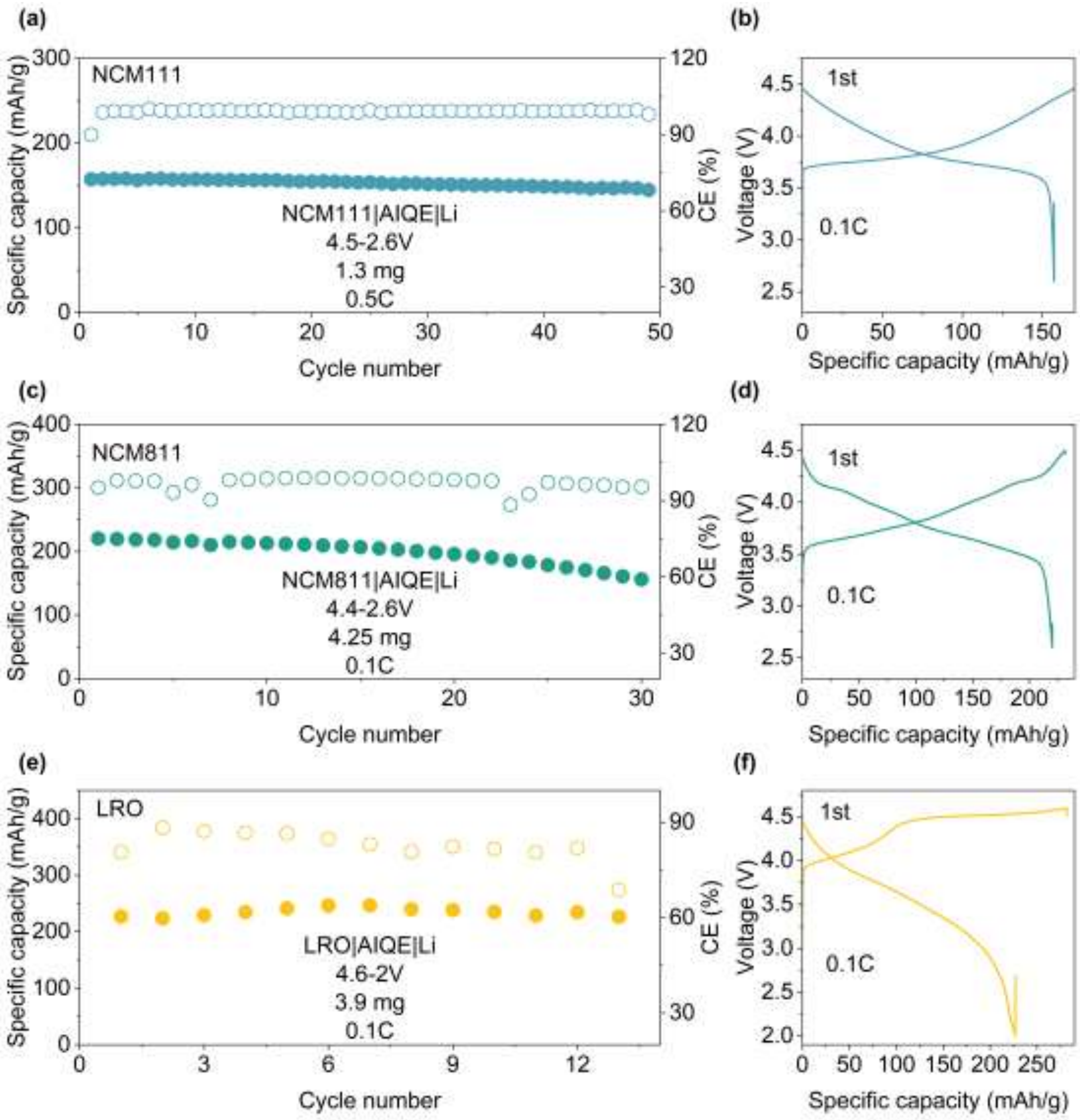

Figure S32. The cycling performance of AIQE based Li metal batteries with NCM111, NCM 811 and LRO as the cathode, respectively.

Table S1 AIQE concentration table

LiFSI concentration	$LiFSI(FEC)_y$
1 m	14.3
7 m	2
15 m	1
20 m	0.7
30 m	0.5
35 m	0.43
40 m	0.37
45 m	0.32
50 m	0.29
55 m	0.27
60 m	0.24

Table S2 Crystalline size calculated by Scherrer equation.

2Theta	Theta	FWHM	Size(nm)
20.9031	10.45155	1.13934	7.09035
22.0324	11.0162	0.2495	32.43858
25.1945	12.59725	0.771924	10.54541
28.028	14.014	1.0123	8.088503
29.7322	14.8661	1.576614	5.213329
35.1529	17.57645	1.103097	7.554487

REFERENCE

1. Hess, B., Kutzner, C., Van Der Spoel, D. & Lindahl, E. GROMACS 4: algorithms for highly efficient, load-balanced, and scalable molecular simulation. *Journal of chemical theory and computation* **4**, 435-447 (2008).
2. Jensen, K.P. & Jorgensen, W.L. Halide, ammonium, and alkali metal ion parameters for modeling aqueous solutions. *Journal of Chemical Theory and Computation* **2**, 1499-1509 (2006).
3. Gouveia, A.S., Bernardes, C.E., Tomé, L.C., Lozinskaya, E.I., Vygodskii, Y.S., Shaplov, A.S., Lopes, J.N.C. & Marrucho, I.M. Ionic liquids with anions based on fluorosulfonyl derivatives: from asymmetrical substitutions to a consistent force field model. *Physical Chemistry Chemical Physics* **19**, 29617-29624 (2017).
4. Dodda, L.S., Cabeza de Vaca, I., Tirado-Rives, J. & Jorgensen, W.L. LigParGen web server: an automatic OPLS-AA parameter generator for organic ligands. *Nucleic acids research* **45**, W331-W336 (2017).
5. Lu, T. & Chen, F. Multiwfn: A multifunctional wavefunction analyzer. *Journal of computational chemistry* **33**, 580-592 (2012).
6. Martínez, L., Andrade, R., Birgin, E.G. & Martínez, J.M. PACKMOL: A package for building initial configurations for molecular dynamics simulations. *Journal of computational chemistry* **30**, 2157-2164 (2009).
7. Berendsen, H.J., Postma, J.v., Van Gunsteren, W.F., DiNola, A. & Haak, J.R. Molecular dynamics with coupling to an external bath. *The Journal of chemical physics* **81**, 3684-3690 (1984).
8. Nosé, S. A molecular dynamics method for simulations in the canonical ensemble. *Molecular physics* **52**, 255-268 (1984).
9. Hoover, W.G. Canonical dynamics: Equilibrium phase-space distributions. *Physical review A* **31**, 1695 (1985).
10. Parrinello, M. & Rahman, A. Polymorphic transitions in single crystals: A new molecular dynamics method. *Journal of Applied physics* **52**, 7182-7190 (1981).
11. Darden, T., York, D. & Pedersen, L. Particle mesh Ewald: An N· log (N) method for Ewald sums in large systems. *The Journal of chemical physics* **98**, 10089-10092 (1993).
12. Lee, C.T., Yang, W.T. & Parr, R.G. DEVELOPMENT OF THE COLLE-SALVETTI CORRELATION-ENERGY FORMULA INTO A FUNCTIONAL OF THE ELECTRON-DENSITY. *Physical Review B* **37**, 785-789 (1988).
13. Henkelman, G. & Jónsson, H. Improved tangent estimate in the nudged elastic band method for finding minimum energy paths and saddle points. *Journal of Chemical Physics* **113**, 9978-9985 (2000).
14. Kresse, G. & Furthmuller, J. Efficient iterative schemes for ab initio total-energy calculations using a plane-wave basis set. *Physical Review B* **54**, 11169-11186 (1996).
15. Kresse, G. & Furthmuller, J. Efficiency of ab-initio total energy calculations for metals and semiconductors using a plane-wave basis set. *Computational Materials Science* **6**, 15-50 (1996).
16. Perdew, J.P., Burke, K. & Ernzerhof, M. Comment on "Generalized gradient approximation made simple" - Reply. *Physical Review Letters* **80**, 891-891 (1998).

17. Song, C., Li, Z., Peng, J., Wu, X., Peng, H., Zhou, S., Qiao, Y., Sun, H., Huang, L. & Sun, S.-G. Enhancing Li ion transfer efficacy in PEO-based solid polymer electrolytes to promote cycling stability of Li-metal batteries. *Journal of Materials Chemistry A* **10**, 16087-16094 (2022).
